# Prediction of Pneumonia in Acute Stroke Patients Using Tongue Pressure Measurements

**DOI:** 10.1371/journal.pone.0165837

**Published:** 2016-11-01

**Authors:** Masahiro Nakamori, Naohisa Hosomi, Kenichi Ishikawa, Eiji Imamura, Takeo Shishido, Tomohiko Ohshita, Mineka Yoshikawa, Kazuhiro Tsuga, Shinichi Wakabayashi, Hirofumi Maruyama, Masayasu Matsumoto

**Affiliations:** 1 Department of Clinical Neuroscience and Therapeutics, Hiroshima University Graduate School of Biomedical and Health Sciences, Hiroshima, Japan; 2 Department of Neurology, Suiseikai Kajikawa Hospital, Hiroshima, Japan; 3 Department of Advanced Prosthodontics, Hiroshima University Graduate School of Biomedical and Health Sciences, Hiroshima, Japan; 4 Department of Neurosurgery, Suiseikai Kajikawa Hospital, Hiroshima, Japan; Osaka University Graduate School of Medicine, JAPAN

## Abstract

Swallowing dysfunction caused by stroke is a risk factor for aspiration pneumonia. Tongue pressure measurement is a simple and noninvasive method for evaluating swallowing dysfunction. We have hypothesized that low tongue pressure may be able to predict pneumonia occurrence in acute stroke patients. Tongue pressure was measured using balloon-type equipment in 220 acute stroke patients. The modified Mann Assessment of Swallowing Ability (MASA) score was evaluated independently on the same day. Tongue pressure was measured every week thereafter. An improvement in tongue pressure was observed within the first 2 weeks. Receiver operating curve analysis was performed to determine the ability of tongue pressure to predict modified MASA score <95, which suggests swallowing dysfunction. The optimal cutoff for tongue pressure was 21.6 kPa (χ^2^ = 45.82, p<0.001, sensitivity 95.9%, specificity 91.8%, area under the curve = 0.97). The tongue pressure was significantly lower in patients with pneumonia than in those without pneumonia. Using a Cox proportional hazard model for pneumonia onset with a cutoff tongue pressure value of 21.6 kPa and adjustment for age, sex, and National Institutes of Health Stroke Scale score at admission, the tongue pressure had additional predictive power for pneumonia onset (hazard ratio, 7.95; 95% confidence interval, 2.09 to 52.11; p = 0.0013). In the group with low tongue pressure, 27 of 95 patients showed improvement of tongue pressure within 2 weeks. Pneumonia occurred frequently in patients without improvement of tongue pressure, but not in patients with improvement (31/68 and 2/27, p<0.001). Tongue pressure is a sensitive indicator for predicting pneumonia occurrence in acute stroke patients.

## Introduction

Swallowing dysfunction is a critical issue in stroke patients. Prevention of aspiration pneumonia and improvement of nutrition are associated with reduced duration of hospitalization and mortality [1–3]. Pneumonia rates have been found to be lower in hospitals using dysphagia screening protocols with appropriate interventions following the identification of patients at risk for aspiration in stroke patients [4].

Among the organs of the oral cavity, the tongue plays a major role in swallowing [5]. The tongue presses the bolus into the pharynx, and it pushes the epiglottis downward, thus closing the larynx. The maximal tongue pressure exerted on the hard palate may offer a quantitative parameter for evaluating tongue motor biomechanics during swallowing [6–8]. Low tongue pressure impairs the control of the bolus, increases the amount of oral residue, which increases the risk of aspiration after swallowing. In addition, low tongue pressure contributes to inadequate closing, which increases the risk of aspiration during swallowing. Several reports have established a relationship between tongue pressure and swallowing dysfunction. Tongue pressure has been associated with the presence of oro-pharyngeal residue using videofluoroscopic examination [9]. In a water-swallowing test, tongue pressure was found to be significantly lower in dysphagic patients than in non-dysphagic patients [10]. Using a non-invasive, easy-to-use tongue pressure measurement tool is an advantage.

The Mann Assessment of Swallowing Ability (MASA) score is an established bedside assessment tool that indicates the risk of swallowing dysfunction in stroke patients [11]. The MASA score consists of 24 items with a total potential score of 200 points. Comparison of the MASA assessment with videofluoroscopic examination has established its reliability among patients with various diseases as well as stroke [12]. The original MASA required a long time and extensive effort for evaluation; therefore, the modified MASA score, which consists of only 12 items with a total potential score of 100 points, was created. It has been reported that patients who score <95 points are at risk for swallowing dysfunction [13].

Swallowing dysfunction is a risk factor for pneumonia, and patients with low MASA score are at a significantly higher risk of aspiration pneumonia and mortality than patients with high MASA score [14]. Knowing the association between tongue pressure and swallowing dysfunction, we hypothesized that low tongue pressure could predict pneumonia onset in acute stroke patients. Predicting pneumonia onset would allow for better patient management and outcomes. In this study, we investigated the association between tongue pressure and pneumonia. Swallowing dysfunction was evaluated using the modified MASA score.

## Materials and Methods

### Ethics Statement

The study protocols were approved by the ethics committee of Suiseikai Kajikawa Hospital and performed according to the guidelines of the national government based on the Helsinki Declaration of 1964. Written informed consent was obtained from all patients or their relatives. All data analyses were blinded.

### Subjects

Consecutive acute stroke patients who were admitted to Suiseikai Kajikawa Hospital from September 1, 2014 to February 28, 2015 were enrolled in this prospective study. We included stroke patients with both ischemic and hemorrhagic stroke who were admitted within 1 week from onset, were aged ≥20 years, and consented to this study (if the patient was not able to consent to the study, consent was obtained from relatives). Some patients had a history of stroke. We excluded patients who were in a coma (the best eye response score in the Glasgow coma scale was 1), underwent craniotomy, or were on mechanical ventilation.

### Data Acquisition

Stroke subtype was determined according to the Trial of Org 10172 in Acute Stroke Treatment criteria [15]. The diagnosis of clinically defined pneumonia was determined based on the agreement of two physicians based on the criteria of the Centers for Disease Control and Prevention [16] as follows. Clinically defined pneumonia criteria require the presence of a new and persistent infiltrate or consolidation on at least 1 chest X-ray or computed tomography examination with 1 of the following clinical signs: fever, leukopenia or leukocytosis, and altered mental status in patients over 70 years of age in the absence of other causes. These should be added to 2 of the following signs: new-onset purulent sputum or change in the character of the sputum, new-onset or progressive cough, rales, and impaired gas exchange. The severity of stroke was evaluated using the National Institutes of Health Stroke Scale (NIHSS) score [17]. Speech-language-hearing therapists and one physician scaled the modified MASA score at the same time with consensus. Clinical technicians measured tongue pressure independently using balloon-type equipment (TPM-01; JMS Co. Ltd., Hiroshima, Japan) on the same day as the evaluation of modified MASA score. Tongue pressure was measured every week thereafter.

The balloon-type equipment consists of a disposable oral probe, an infusion tube as a connector, and a recording device. For tongue pressure measurement, the subjects were placed in a relaxed sitting position and asked to place the balloon in their mouths, holding the plastic pipe at the midpoint of their central incisors with closed lips. The subjects were asked to maintain this position as clinicians adjusted the probe and confirmed that it was in the correct position. The subjects were then asked to raise their tongue and compress the small balloon with their palate at maximum voluntary effort for 7 seconds as described previously [7,18]. This measurement was performed 3 times with the subjects resting for approximately 30 seconds and rinsing the mouth between each measurement. The maximum value from the 3 measurements was defined as the tongue pressure for each subject.

The reliability of intraindividual measurement was previously reported [19]. In this study, we reconfirmed the reliability of tongue pressure measurement. We measured normal subjects three times per day, and the highest value from the 3 measurements was defined as the tongue pressure. These measurements were repeated for ten days, and the resulting coefficient of variation was 5.64%.

The modified MASA score and tongue pressure measurements were evaluated in the sitting position. These evaluations were performed every week until the patients were discharged from the hospital. The NIHSS score, tongue pressure, and modified MASA score were evaluated by different physicians or therapists who were blinded to the other findings.

We monitored patients for signs of pneumonia during the 30 days following admission. If patients were discharged earlier than the 30th day, we monitored the patients until the day of discharge.

### Statistical Analysis

The data were expressed as the mean ± standard deviation or the median (minimum, maximum) for continuous variables and frequencies and percentages for discrete variables. Statistical analysis was performed using JMP 11 statistical software (SAS Institute Inc., Cary, NC, USA). The statistical significance of intergroup differences was assessed using *t-*tests or χ^2^ tests as appropriate. The baseline data in the stroke patients were analyzed, and two-step strategies were employed to assess the relative importance of variables in their association with tongue pressure using least square linear regression analysis. First, a univariate analysis was performed. Then, a multi-factorial analysis was performed with selected factors that had p<0.20 on univariate analysis. Receiver operating characteristic (ROC) analysis was performed to determine the tongue pressure predicting a modified MASA score <95, which suggests swallowing dysfunction. Kaplan–Meier and Cox proportional hazard regression analyses were performed to test the difference in the development of pneumonia between the high and low tongue pressure groups. A univariate split-plot approach was utilized for changes in tongue pressure over time, with the last observation carried forward for missing data. We considered p<0.05 as statistically significant.

## Results

During the study period, 296 stroke patients were admitted in our hospital. Nineteen patients or their relatives did not consent to this study, 19 patients were admitted >1 week after onset, 30 patients were in a coma, and 8 patients underwent craniotomy. Therefore, 220 patients were included in this study. The subjects’ backgrounds and characteristics at the time of enrollment are shown in Table 1. The number of patients with a modified MASA score <95 was 98 (44.5%). The mean maximum tongue pressure was 22.8±14.6 kPa.

**Table 1:** Patient characteristics.

	All	Tongue pressure<21.6 kPa	Tongue pressure≥21.6 kPa	p–value
Factors	n = 220	n = 95	n = 125	
Body height, m	1.59 ± 0.10	1.56 ± 0.10	1.61 ± 0.09	<0.001
Body weight, kg	58.3 ± 13.5	54.6 ± 13.0	61.1 ± 13.3	<0.001
Age, year	73.9 ± 12.4	78.5 ± 12.6	70.4 ± 11.2	<0.001
Women, n (%)	88 (40.0%)	47 (49.5%)	41 (32.8%)	0.012
Subtypes				<0.001
ATBI, n (%)	40 (18.2%)	15 (15.8%)	25 (20.0%)	
CEI, n (%)	49 (22.3%)	30 (31.6%)	19 (15.2%)	
LI, n (%)	41 (18.6%)	10 (10.5%)	17 (13.6%)	
Others, n (%)	48 (21.8%)	15 (15.8%)	31 (24.8%)	
ICH, n (%)	42 (19.1%)	25 (26.3%)	33 (26.4%)	
Hypertension, n (%)	170 (77.3%)	70 (73.7%)	100 (80.0%)	0.268
Diabetes mellitus, n (%)	72 (32.7%)	38 (40.0%)	34 (27.2%)	0.045
Dyslipidemia, n (%)	97 (44.1%)	35 (36.8%)	62 (49.6%)	0.059
Atrial fibrillation, n (%)	58 (26.4%)	35 (36.8%)	23 (18.4%)	0.002
Tongue pressure, kPa	22.8 ± 14.6	8.8 ± 7.7	33.4 ± 8.3	<0.001
modified MASA score	97 (33, 100)	85 (33, 99)	99 (74, 100)	<0.001
modified MASA score<95, n (%)	98 (44.5%)	90 (94.7%)	8 (6.4%)	<0.001
NIHSS score	4 (0, 31)	9 (1, 31)	2 (0, 15)	<0.001
Pneumonia onset, n (%)	35 (15.9%)	33 (34.7%)	2 (1.6%)	< 0.001

MASA, Mann Assessment of Swallowing Ability; NIHSS, National Institutes of Health Stroke Scale; ATBI, atherothrombotic brain infarction; CEI, cardiogenic embolism infarction; LI, lacunar infarction; ICH, intracerebral hemorrhage

Modified MASA and NIHSS scores are expressed as the median (minimum, maximum).

**Fig 1:**
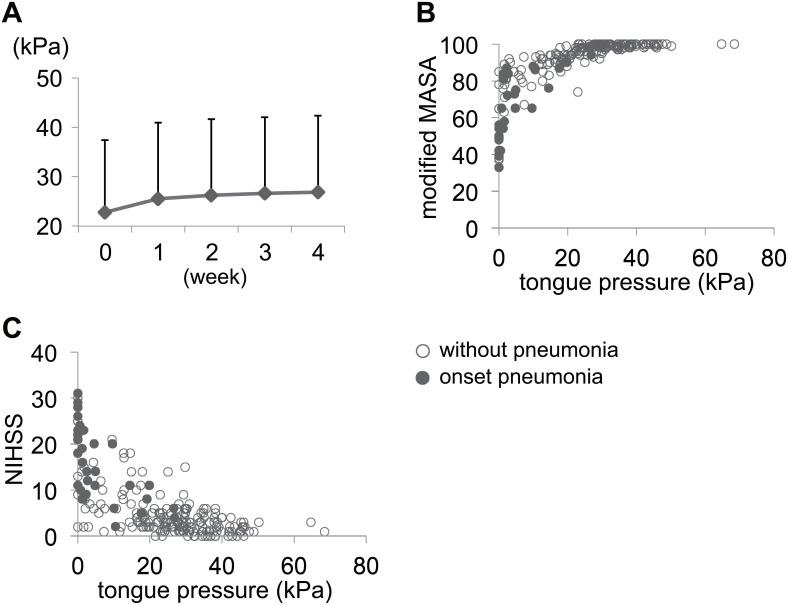
Tongue pressure time course and the association of tongue pressure with modified MASA and NIHSS scores. The patients showed a significant increase in tongue pressure with repeated measurements during the observation period. The plot shows the mean values and the error bars indicate the standard deviation (A). An improvement in tongue pressure was observed over the first 2 weeks of hospitalization. Scatter diagram of tongue pressure with modified MASA score (B) and NIHSS score (C) at admission is shown. Modified MASA score increased with tongue pressure elevation curvilinearly, and reached a stable state at a tongue pressure >21.6 kPa. The NIHSS score decreased with tongue pressure elevation but varied widely compared with the modified MASA score shown in the scatter diagram. ○, without pneumonia; ●, onset pneumonia.

We evaluated the change of maximum tongue pressure every week (Fig 1A). An improvement in tongue pressure was observed within the first 2 weeks. We compared tongue pressure of the first measurement to that of 2 weeks after. The tongue pressure was significantly increased during the first 2 weeks using paired *t*-test (p<0.001). In addition, the patients showed a significant increase with repeated measurements in tongue pressure during the observation period (timing, F = 115.96; p<0.001; power = 1.00; adjusted R^2^ = 0.908; F_220, 413_ = 29.37; p<0.001; n = 220).

Potential factors associated with tongue pressure at admission were evaluated from the factors listed in Table 1. In this analysis, age, stroke type (lacunar infarction or intracerebral hemorrhage), and modified MASA score were determined to be independent factors significantly associated with tongue pressure (Table 2). The optimal cutoff for tongue pressure to predict a modified MASA score <95 was 21.6 kPa from the ROC analysis (χ^2^ = 45.82, p<0.001, sensitivity 95.9%, specificity 91.8%, AUC = 0.97). Scatter diagrams for the association of tongue pressure with modified MASA score and NIHSS score are shown in Fig 1B and 1C. The modified MASA score increased with tongue pressure elevation and reached a maximum at tongue pressures higher than 21.6 kPa. The NIHSS score decreased with tongue pressure elevation.

**Table 2:** Factors influencing tongue pressure.

Factor	Univariate analysis	Multivariate analysis			
	p value	predictor	95% CI		p-value
Body height	<0.001	2.95	-19.9	25.7	0.80
Body weight	<0.001	0.044	-0.087	0.176	0.51
Age	<0.001	-0.21	-0.33	-0.086	<0.001
Sex	<0.001	0.56	-1.43	2.54	0.58
Subtype	<0.001				
ATBI		0.48	-2.20	3.17	0.72
CEI		0.18	-3.39	3.75	0.92
LI		3.29	0.48	6.10	0.02
Others		1.00	-	-	-
ICH		-3.08	-5.80	-0.37	0.03
Hypertension	0.33				
Diabetes mellitus	0.04	-0.28	-1.73	1.17	0.70
Dyslipidemia	0.04	-0.42	-1.78	0.93	0.54
Atrial fibrillation	<0.001	-0.25	-2.36	1.85	0.81
modified MASA score	<0.001	0.55	0.37	0.72	<0.001
NIHSS score	<0.001	-0.10	-0.51	-030	0.61

CI, confidence interval; ATBI, atherothrombotic brain infarction; CEI, cardiogenic embolism infarction; LI, lacunar infarction; ICH, intracerebral hemorrhage MASA, Mann Assessment of Swallowing Ability; NIHSS, National Institutes of Health Stroke Scale

**Fig 2:**
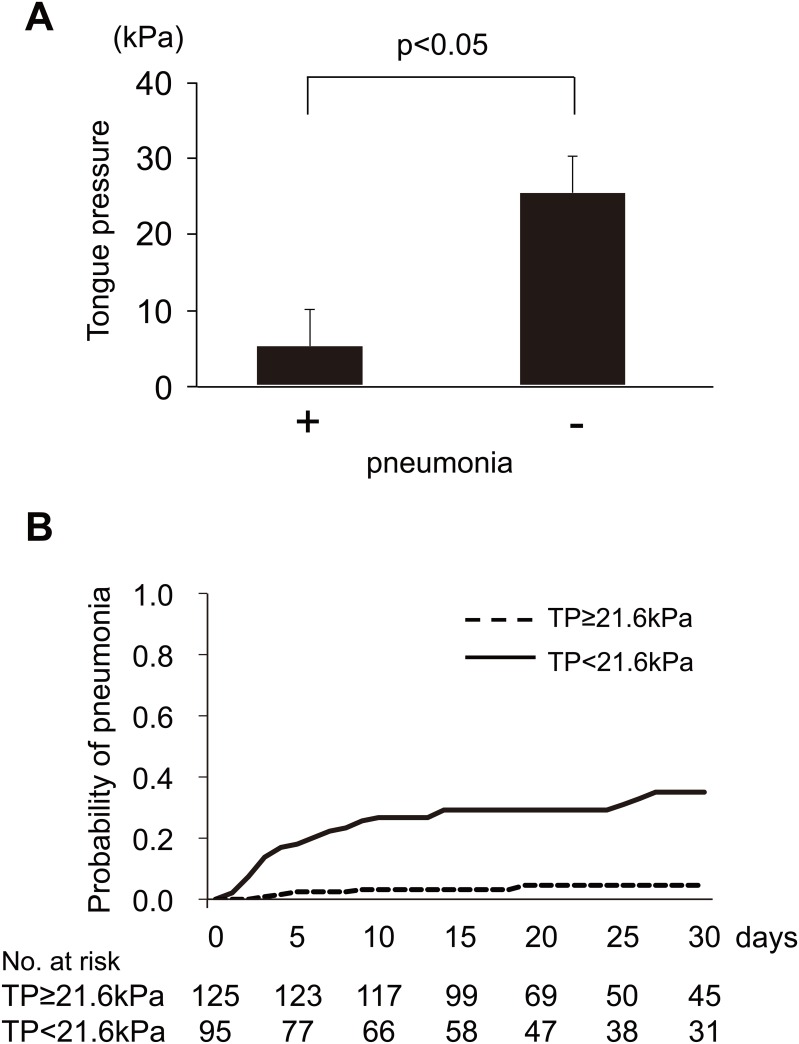
Relationship between tongue pressure and pneumonia. The tongue pressure was significantly lower in patients with pneumonia than in those without pneumonia using unpaired *t*-tests. The error bars indicate the standard deviation (A). Kaplan–Meier curves of the duration until the development of pneumonia between the high tongue pressure group (≥21.6 kPa) and low tongue pressure group (<21.6 kPa) (B). The low tongue pressure (TP) group had a higher incidence of pneumonia.

Thirty-five subjects developed pneumonia during the observation period. No patient had pneumonia before stroke onset. We compared the maximum tongue pressure at admission between patients with and without pneumonia. The tongue pressure was significantly lower in patients with pneumonia than in those without pneumonia (Fig 2A). The results of the Kaplan–Meier analysis to compare the development of pneumonia between the high (≥21.6 kPa) and low (<21.6 kPa) tongue pressure groups are shown in Fig 2B. The low tongue pressure group had a significantly higher incidence of pneumonia than the high tongue pressure group (p<0.001, log-rank test). Cox proportional hazard model for pneumonia onset was performed with a tongue pressure cut-off value of 21.6 kPa adjusting for age and sex. The tongue pressure was found to be independently associated with pneumonia onset (hazard ratio, 17.27; 95% confidence interval, 5.10 to 107.86; p<0.001; model 1 adjusted for age and sex). Additionally, we added the NIHSS score to the adjustment. Even after adjustment with these factors, the tongue pressure was independently associated with pneumonia onset (hazard ratio, 7.95; 95% confidence interval, 2.09 to 52.11; p = 0.0013; model 2 adjusted with age, sex, and NIHSS score).

In the group with low tongue pressure, 27 of 95 patients showed improvement of tongue pressure up to 21.6 kPa within 2 weeks from admission. Only two of them (7.4%) developed pneumonia during the observational period. In contrast, in the non-improved patients, 31 out of 68 patients (45.6%) developed pneumonia. There was a significant difference in pneumonia onset between the patients with improvement and those with no improvement (p<0.001, with the Cox proportional hazard model).

## Discussion

In this study, we measured tongue pressure in constitutive acute stroke patients using balloon-type equipment and found that it was significantly associated with the modified MASA score, which has been established as a reliable bedside assessment for detecting swallowing dysfunction [13]. We also showed that lower tongue pressure (<21.6 kPa) was an independent risk factor for pneumonia. Moreover, tongue pressure was evaluated repeatedly and was found to improve over time, especially over the first 2 weeks of admission.

Tongue pressure has been reported as low on the paralyzed side using sensor sheets in acute stroke patients [10]. In this method, a sensor sheet is attached to the hard palate of patients, and a water-swallowing test is performed. An advantage of this method is the ability to evaluate the focal weak point of the tongue. However, it is more invasive than the balloon-based method used in the present study. There are mainly three types of balloon-based tongue pressure evaluating equipment: the Kay Pentax device, the Iowa Oral Performance Instrument (IOPI) device, and the JMS device. These balloon-based methods enable us to evaluate tongue pressure non-invasively and repeatedly. The JMS device show lower values than the other balloon-based devices, but the values can be correlated linearly [7]. The JMS probe is fixed onto the incisor teeth, and one can measure tongue pressure at that same position.

The MASA score is a reliable tool for evaluating the risk of swallowing dysfunction and can be used at the patient’s bedside with no special instruments [12,13,20]. However, this non-invasive screening test requires approximately 15 minutes to complete when performed by a skilled evaluator. The modified MASA score is simplified from the original version, but it is still subjective and requires some training and experience. In this study, the modified MASA score was evaluated by skilled speech-language-hearing therapists and compared to the tongue pressure. The measurement of tongue pressure is simple and objective, requires less training, and can be completed within 3 minutes.

In this study, the modified MASA score increased according to the tongue pressure and reached a maximum at a tongue pressure >21.6 kPa. Among Japanese healthy subjects, using the same balloon-based equipment, the standard maximum tongue pressure was found to be reduced with aging (41.7±9.7 kPa in subjects their 20s and 31.9±8.9 kPa in subjects in their 70s [6]. In the studies using the same balloon-based equipment, Japanese elderly subjects with frailty showed a maximum tongue pressure of 18.0±12.0 kPa [8,21]. Additionally, in patients with spinal and bulbar muscular atrophy, the maximum tongue pressure of patients with severe dysphagia was observed to be approximately <20 kPa [22]. The numerical value suggesting a risk of swallowing dysfunction identified in our study was consistent with this previous report. Additionally, these maximum tongue pressure values were lower than those of the healthy subjects [6]. However, to the best of our knowledge, no previous studies have measured the tongue pressure in acute stroke patients using balloon-based equipment. Moreover, no other report has evaluated the relationship of tongue pressure with modified MASA or NIHSS scores. As shown in Fig 1B and 1C, both showed floor or ceiling effects when tongue pressure reached around 20 kPa. It showed the patient with full modified MASA score and low NIHSS score shows tongue pressure greater than around 20 kPa. It is interesting to note that swallowing dysfunction hardly emerged above 20 kPa of tongue pressure. Physiological research is needed to understand the underlying reasons.

The tongue pressures were significantly lower in patients with pneumonia than in patients without pneumonia. Low tongue pressure is considered to be a risk factor for pneumonia. The hazard ratio of pneumonia onset reveals that patients with low tongue pressure were at higher risk for pneumonia, even after adjusting for the severity of stroke (NIHSS score at admission). The relationship between low tongue pressure and pneumonia onset might be explained by the presence of swallowing dysfunction.

Tongue pressure improved favorably over the first 2 weeks of admission in this study. The onset rate of pneumonia was significantly higher in the group without improvement of tongue pressure than in the group with tongue pressure improvement. It has also been reported that tongue exercise in stroke patients significantly increases tongue pressure and reduced penetration [23]. In this study, all subjects underwent rehabilitation as appropriate. Especially, swallowing training was performed intensively in the patients who showed swallowing dysfunction. Rehabilitation could have affected the results. Rehabilitation towards an increase in tongue pressure may improve swallowing function, thus decreasing the risk of pneumonia. Tongue pressure and its recovery may thus be a replicable and objective tool for predicting pneumonia occurrence. However, it is not clear which type of rehabilitation is effective to improve swallowing function. Further prospective studies are needed to evaluate the influence of swallowing training on increased tongue pressure.

This study had several limitations. First, the tongue pressure may not be accurately evaluated in patients with a disturbance of consciousness, aphasia, or attention disorders. Regardless of tongue function, these conditions usually increase the risk of aspiration and pneumonia. In this study, we excluded patients in a coma, but included patients with a mild disturbance of consciousness, aphasia, or attention disorders, which limited the potential for selection bias. The modified MASA score includes factors such as consciousness, aphasia, and communication ability. Tongue pressure may decrease in association with a low modified MASA score and thus reflect the risk of swallowing dysfunction. A hybrid evaluation of modified MASA scores and tongue pressures may improve the assessment of swallowing function. Second, tongue pressures cannot reflect all functions of swallowing. Videofluoroscopic examination has been used for evaluating aspiration but has limitations, such as exposure to radiation and difficulties in conducting examinations in disabled patients and analyzing the biomechanics of swallowing organs [24]. Additionally, it is not possible to perform videofluoroscopic examinations for all acute stroke patients. Therefore, it is important that the risk of swallowing dysfunction be accurately evaluated using a combination of simple modalities such as ultrasonography for bedside assessment [25].

## Conclusions

Tongue pressure is a sensitive indicator for predicting pneumonia in acute stroke patients. Therefore, as a bedside assessment tool, tongue pressure measurements may be helpful for evaluating swallowing function.
